# Genome Wide Association Mapping of Root Traits in the Andean Genepool of Common Bean (*Phaseolus vulgaris* L.) Grown With and Without Aluminum Toxicity

**DOI:** 10.3389/fpls.2021.628687

**Published:** 2021-06-25

**Authors:** Daniel Ambachew, Matthew W. Blair

**Affiliations:** Department of Agricultural and Environmental Sciences, Tennessee State University, Nashville, TN, United States

**Keywords:** abiotic stress, citrate and malate transporters, hydroponic culture, root traits, single nucleotide polymorphism markers

## Abstract

Common bean is one of the most important grain legumes for human diets but is produced on marginal lands with unfavorable soil conditions; among which Aluminum (Al) toxicity is a serious and widespread problem. Under low pH, stable forms of Al dissolve into the soil solution and as phytotoxic ions inhibit the growth and function of roots through injury to the root apex. This results in a smaller root system that detrimentally effects yield. The goal of this study was to evaluate 227 genotypes from an Andean diversity panel (ADP) of common bean and determine the level of Al toxicity tolerance and candidate genes for this abiotic stress tolerance through root trait analysis and marker association studies. Plants were grown as seedlings in hydroponic tanks at a pH of 4.5 with a treatment of high Al concentration (50 μM) compared to a control (0 μM). The roots were harvested and scanned to determine average root diameter, root volume, root surface area, number of root links, number of root tips, and total root length. Percent reduction or increase was calculated for each trait by comparing treatments. Genome wide association study (GWAS) was conducted by testing phenotypic data against single nucleotide polymorphism (SNP) marker genotyping data for the panel. Principal components and a kinship matrix were included in the mixed linear model to correct for population structure. Analyses of variance indicated the presence of significant difference between genotypes. The heritability of traits ranged from 0.67 to 0.92 in Al-treated and reached similar values in non-treated plants. GWAS revealed significant associations between root traits and genetic markers on chromosomes Pv01, Pv04, Pv05, Pv06, and Pv11 with some SNPs contributing to more than one trait. Candidate genes near these loci were analyzed to explain the detected association and included an Al activated malate transporter gene and a multidrug and toxic compound extrusion gene. This study showed that polygenic inheritance was critical to aluminum toxicity tolerance in common beans roots. Candidate genes found suggested that exudation of malate and citrate as organic acids would be important for Al tolerance. Possible cross-talk between mechanisms of aluminum tolerance and resistance to other abiotic stresses are discussed.

## Introduction

Common bean (*Phaseolus vulgaris*) is one of the most important grain legumes for human diets (Blair, 2013). The legume is produced on marginal lands in many parts of the globe where unfavorable soil factors constrain yield potential (Eticha et al., 2010; Yang et al., 2013). Among these factors, soil acidity is an important problem of crop production in the humid and semi-humid sub-tropical or tropical regions of bean production (Rangel et al., 2010). These acid soils represent 30–50% of the world’s arable land (Hede et al., 2001; Kochian et al., 2004). Under low pH stable forms of Aluminum (Al) are solubilized into phytotoxic Al^3+^ ions (Bojórquez-Quintal et al., 2017). Therefore, Al toxicity is one of the major problems of common bean production in low pH soils typical of Latin America and African production zones (Rangel et al., 2010). Additionally, nutrient deficiencies, and other proton and mineral toxicities are the important factors limiting crop production on acidic soils (Eticha et al., 2010; Zhou et al., 2011).

Al is the most abundant metal in earth’s crust (Arunakumara et al., 2013; Gupta et al., 2013)and as mentioned is one of the major constraints to crop production on most acidic soils (Barceló and Charlotte, 2002). Under acidic conditions, when the soil pH is below 4.5, Al dissolves rapidly into soil solution and becomes a positive ion [Al (H_2_O)_6_]^3+^, generally referred to as soluble aluminum (Al^3+^) (Delhaize et al., 1993; Kochian et al., 2005). High concentration of Al^3+^ is highly toxic to plants and primarily inhibit root growth and function (Arunakumara et al., 2013; Mendoza-Soto et al., 2015). Al toxicity causes 30 – 60% yield reduction and substantial economic loss in common bean (Horst et al., 2009; Rangel et al., 2010). Even a micro-molar concentration of Al^3+^, can interfere with many physiological and cellular processes in susceptible plants (Hede et al., 2001; Kochian et al., 2004).

Aluminum toxic conditions inhibit the growth and function of roots when the Al^3+^ ions injure the root apex, which in turn affects the function of other plant parts (Rangel et al., 2007, 2010; Yang et al., 2013, 2011). While primary physiological response is at the root level Al toxicity results in strong yield reduction (Gupta et al., 2013). Poor root growth, changed root morphology, stunted plant growth, thicker lateral and taproots, browning of roots, inefficient water and nutrient uptake, and accumulation of Al and other toxic ions (Mugai et al., 2000; Bartoli et al., 2017), are symptoms of Al toxicity induced in susceptible lines of common bean (Rao et al., 2008, 2016).

Tolerant plants can be developed by different mechanisms of response to Al toxicity (Bojórquez-Quintal et al., 2017). The reaction of Al tolerance has been studied in many plant species and external and internal mechanisms have been identified (Hede et al., 2001). An external tolerance mechanism is where plants exclude Al^3+^ from the root apex using selective permeability of the plasma membrane or by exudation of organic acids, root mucilage or free phosphate to bind the Al ions. The internal tolerance mechanisms that confer the ability to tolerate Al in the plant symplasm use Al binding proteins (Gupta et al., 2013). Both mechanisms are related to mitochondrial metabolism and acid transport (Nunes-Nesi et al., 2014). Though rehabilitation is only on the thin upper layer of the soil surface, Al toxicity and soil acidity can be corrected with the bulk application of lime or gypsum (Neto et al., 2020). This type of soil amelioration will restrict the plant roots from growing deeper which limits their ability to access and extract water from deeper soils and make plants prone to drought stress. Long-term sustainable agricultural production requires the use of Al tolerant genotypes improved through plant breeding combined with the application of adapted agronomic practices.

Sources of genetic resistance/tolerance to Al toxicity in common beans are multiple: Blair et al. (2009), identified common bean genotypes from an Andean gene pool that are tolerant to a higher level of (20 μM) Al concentration. Butare et al. (2011) reported on inter-specific lines combining Al^3+^ and drought resistance/tolerance from a set 11 bean genotypes from *P. vulgaris*, *P. coccineus*, and *P. acutifolius*. Butare et al. (2012) developed recombinant inbred lines (RILs) for Al^3+^ tolerance from the interspecific cross between SER16 (sensitive) and G35246-Q (tolerant). SER16 is common bean breeding line with ability to remobilize photosynthate while G35246-Q is an accession of *P. coccineus* tolerant to Al toxicity. Most research was with Mesoamerican small-seeded beans like SER16. However, Al tolerance mechanisms were also characterized using a cross of Al-tolerant Andean genotype ICA Quimbaya and Al-susceptible Mesoamerican genotype, VAX-1 (Yang et al., 2013). Al tolerance in common beans involves Al-activated exudation of organic acids with malate, citrate and oxalate anions differentially produced in Al tolerant versus sensitive genotypes (Miyasaka et al., 1991; Mugai et al., 2000; Yang et al., 2000; Eticha et al., 2010; Rangel et al., 2010).

Common bean plants have evolved various additional adaptive strategies to alleviate and recover from the adverse effects of Al stress or other abiotic stresses. Root system architecture is very responsive to soil elements and enables the plants to avoid environmental challenges and abiotic stresses by sensing and responding to them (Pandey et al., 2017). Root metabolites (Eticha et al., 2010; Rangel et al., 2010), root growth rate, the average root diameter, total root length and density of primary and lateral roots are phenotypes frequently used to measure the effects on roots of Al toxicity, drought or salinity stress and phosphorous deficiency (Yan et al., 2004; Beebe et al., 2006; Ochoa et al., 2006; Asfaw and Blair, 2012).

The genetic control and inheritance of Al resistance have not been well studied in common beans except for bi-parental QTL mapping (López-Marín et al., 2009). That pioneering study tried to understand the genetic architecture of Al resistance and showed the polygenic inheritance of Al resistance in common beans. The authors reported 9 and 7 Quantitative Trait Loci (QTLs), respectively, for root traits from the evaluation of RILs derived from the cross between DOR364 and G19833 in Al-treatment and control growth conditions. Relative traits were also calculated from comparison of +/− Al. In this population, DOR364 was a Mesoamerican small seed red bean and G19833 was a large-seeded Andean, but both parents were tolerant to Al toxicity. The same study reported that the identified QTLs were distributed all over the genome except chromosome Pv10 and QTLs with major effect resided on Pv09.

In a later study, Njobvu et al. (2020), identified eight QTLs for root length and root dry weight from the evaluation of 150 F4:5 recombinant inbred lines (RILs) derived from a cross between Solwezi, a landrace, with type IV growth habit and resistant to Al toxicity and AO-1012-29-3-3A, a determinate Andean dark red kidney variety with susceptibility to Al toxicity. The QTL identified by this group resided on Pv02, Pv04, Pv06, Pv07, Pv09, and Pv10. Colocalization of QTL for Al resistance and tolerance to phosphorous deficiency was also reported suggesting the cross link in bean roots between the two conditions of acidic soils (López-Marín et al., 2009). Most other studies of Al toxicity tolerance in common beans have been physiological in nature (Rangel et al., 2007; Mimmo et al., 2009) but some have looked at germplasm screening (Blair et al., 2009).

Association mapping exploits the linkage disequilibrium (LD) present among individuals from natural populations or germplasm collections to dissect the genetic basis of complex trait variation (Myles et al., 2009). Germplasm collections generally contain more genetic diversity than segregating progenies and, since association mapping exploits all the recombination events that have occurred in the evolutionary history of the association panel, a much higher mapping resolution is expected (Ingvarsson and Street, 2011). In addition, the number of QTLs that can be mapped for a given phenotype is not limited to the segregation products in a specific cross, but rather by the number of QTLs underlying the trait and the degree to which the studied population captures the genetic species-wide diversity (Yano et al., 2016).

Recently, a number of genome wide association studies (GWAS) have been conducted on common beans for diseases like *Rhizoctonia solani* (Oladzad et al., 2019b); angular leaf spot (Perseguini et al., 2016); common bacterial blight (Shi et al., 2011), nematodes (Wen et al., 2019); anthracnose (Zuiderveen et al., 2016); heat and drought stresses (Oladzad et al., 2019a), agronomic traits (Kamfwa et al., 2015a, b; Moghaddam et al., 2016; Raggi et al., 2019), cooking time and culinary quality traits (Cichy et al., 2015b) using diversity panels either from one or both the Mesoamerican and Andean genepool. One neglected area of study is the root traits of diverse genotypes and this is especially the case for Al toxicity effects on legumes such as common bean, the leading pulse for direct human consumption.

The major goal of this study was to identify candidate genes associated with Al tolerance in early stages of development in common beans. Our hypothesis was that in a hydroponic system with careful control of aluminum stress levels we could find repeatable phenotypic differences of high heritability for the root traits measured which would allow the identification of candidate genes through a genome wide association study approach. We used common bean seedlings, heritability estimates and repeated measures to accommodate and study the large number of genotypes of an Andean genepool panel, as this class of large-seeded common beans has been less well studied for Al toxicity tolerance but is grown in many regions of the world where low pH soils are prevalent, such as East and Southern Africa. Seedlings present the first response to the serious soil challenge of high Al concentrations and are critical for plant establishment.

## Materials and Methods

### Plant Materials

A subset of 227 genotypes from the Global Andean bean diversity panel (ADP) of 278 cultivars (Supplementary Table 1), were phenotyped for root traits using a hydroponic system described below. The ADP consisted of cultivated genotypes only. The geographic sources of the varieties were Africa and North America. The panel was assembled mainly from public and private breeding programs, including varietal releases, elite lines, and land races (Cichy et al., 2015a). The ADP represented different market classes, seed size and three growth types (I, II, and III). For each genotype, seeds were surface sterilized for 1 min with 70% alcohol, 2% sodium hypochlorite (NaClO) and rinsed with deionized water and dried with a sterilized paper towel. Seeds were then scarified using scalpel just on the opposite side of the seed micropyle to ensure uniform germination of the genotypes from the panel. After scarification, the seed were transferred to a sterilized magenta box with a sterile paper towel in it. Deionized water was added to moisten the paper.

### Hydroponic System

After germination at three days, seedlings with uniform length were transferred and planted into a hydroponic system with a standard protocol for Al toxicity testing and plant nutrient solution based on previous studies (Rao et al., 2008; Butare et al., 2011). Plants were grown as seedlings in hydroponic tanks at a pH of 4.5 with a treatment of high Al concentration (50 μM) compared to a control (0 μM). The seedlings were individually placed into 5 cm diameter × 5 cm deep net plastic pots and suspended over 30.2 cm deep, 65.4 cm long × 43.8 cm wide, 50-liter Sterilite^®^ black plastic tanks. Each tank was set up with a continuous aeration system with two eight port Hydrofarm ActiveAqua^TM^ air pumps and the constant pumping of nutrient solution with 172 GPH EcoPlus^®^ Adjustable flow submersible water pumps to ensure water agitation and avoid sedimentation. The experimental setup was as shown in Supplementary Figure 1 for arrangement of hydroponic tanks in the control and Al treatments along with recirculating pumps and chambers as well as aeration system. The number of tanks used per treatment is also shown as well as the method for sensing solution electrical conductivity (EC), its pH and temperature.

### Nutrient Solution

The standard nutrient solution was the same for the two treatments except in concentration of Al (50 μM versus 0 μM) added as Al chloride (AlCl_3_). The remaining nutrients were 286 μM CaSO_4_.2H_2_O, 300 μM KNO_3_, 150 μM NH_4_NO_3_, 2.5 μM NaH_2_PO_4._H_2_O, 150 μM MgCl_2_.6H_2_O, 14 μM CaCl_2_.H_2_O, 5 μM FeCl_3_.6H_2_O, 5 μM Na_2_EDTA.2H_2_O, 1 μM MnCl_2_.4H_2_O, 1 μM ZnCl_2_, 0.2 μM CuCl_2_.2H_2_O, 6H_3_BO_3_, 5 μM NaSiO_3_.9H_2_O, 0.001 μM NaMoO_4_.2H_2_O, 57.5 μM NaCl (Rao et al., 2008). The pH, Electrical conductivity (EC) and temperature of the hydroponic tank system and around the roots were monitored in real time using a Supervisory Control and Data Acquisition (SCADA) system designed by indoor grower’s world, Nashville, TN. The system consisted of a master site controller and two reservoir remote terminal units (RTUs). A randomized balanced design was used with three replicates, reusing the same hydroponic tanks over three planting times, with bleach sterilization (7.5% Sodium Hypochlorite) between uses.

### Greenhouse Conditions

A glass-roofed greenhouse at the Tennessee State University Agricultural Research and Extension Center (AREC) was the site of the benches used to hold the hydroponic system 1.5 m above a gravel floor. Temperature conditions were set to 23 ±3°C (day) / 20 ± 3°C (night) with relative humidity (RH) between 70 and 80%. The greenhouse temperature and relative humidity was monitored with *WADSWORTH*^®^ step up control system, and when heating was needed it was provided by a natural gas heater with winter shade cloth for heat retention. Fans were used for air circulation. The first replication of the experiment was planted in September, the second in October and the third replication was planted in November 2018. Each replication took 15 days from seeding to root image acquisition. Upon planting, each tank was covered with a 5 cm thick Styrofoam floating sheet cut to the size of the tank and able to carry 49 seedlings in a 5 × 10-hole design where one of the holes was used to insert air sones, hydroponic solution control system probes.

### Root Phenotyping

Phenotypic data were measured on the seedlings growing as described above in the hydroponic tanks. The small plants were carefully removed from the tank and the float collar and the root systems scanned with a flatbed EPSON perfection V850 pro scanner (Seiko EPSON Corporation, Japan). The following traits were measured: (1) Average root diameter (AvgD), (2) Number of root forks (NRF), (3) Number of root tips (NRT), (4) Number of root links (NRL), (5) Root surface area (RSA), (6) Root volume (RV), (7) Root surface area (RSA), and (8) Total root length (TRL), were recorded by. Scanned images of the harvested seedling roots were analyzed using a software program WinRHIZO pro V 2008b (Regent Instruments, Inc., Quebec, Canada). The root images were acquired in a gray scale to a resolution of 800 dots per inch (dpi). The analysis was done on the root morphology by setting the rough edge and noise removal to higher level, and dark root on white background measurement option.

### Analyses of Variance and Adjusted Means Estimates

Analyses of variance was conducted on the recorded root data on each treatment; Al and control treatment using mixed models in SAS 9.3 (SAS Institute Inc, 2011), using the following equation:

Y=ijμ+Tt+ibj+e;ij

where *Yij* is a response variable of genotypes the i^*th*^ genotype in j^*th*^ replication, μ and T_*i*_ are fixed parameters such that the mean for the *i*^*th*^ genotype is μ_*i*_ = μ + *T*_*i*_, *b*_*j*_ is the random effect associated with the *j*^*th*^ replication and *e*_*ij*_ is the random error associated with the genotypes in each replication. All the datasets were filtered for outlier genotypes that violate the assumption of the ANOVA even after data transformation. To estimate the adjusted means based on best linear unbiased estimation, we fit the genotypes as fixed and replication as random effects in the model. The variance components and the adjusted means based best linear unbiased estimation were estimated by fitting genotypes and replication as random in the mixed model. Heritability of traits was calculated for each treatment and combined data using the method described in Holland et al. (2003). The adjusted means from the best linear unbiased estimates were then used to calculate the percent change in the traits from control treatment and Al -treatment using the following formula:

Percentchangey=i((YCi-YAitreatment)YCi)x100%

where Y_*i*_ is the percent change of a trait of the i^*th*^ genotype, YC_*i*_ is the mean value of a trait for the i^*th*^ genotype, and YA is the mean value of a trait for the i^*th*^ genotype in the Al treatment. The percent change of means of traits were used for genome wide association. Similarly, the adjusted means based of prediction were used for genomic prediction analysis.

### Genotyping Quality Control

Previous genotypic data on the ADP including 31K SNPs found by ApeKI genotyping-by-sequencing (GBS) method described by Elshire et al. (2011) optimized for common beans by Hart and Griffiths (2015). The associated HapMap file of the SNP dataset were publicly available at the feed the future website^1^. After filtering for missingness >10%, SNPs with missing data were imputed using the LD KNNi imputation method (Money et al., 2015) plugin of TASSEL software (Bradbury et al., 2007) with the default parameters. Two SNP data sets were generated from the original 31K SNP dataset. the first dataset (DATASET1) was a set of 13906 SNPs and 227 genotypes which were retained after SNPs with MAF < 0.05, heterozygosity >0.02 and SNPs with more than two alleles were filtered out. The second dataset (DATASET2). A set of 2286 SNP markers and 227 genotypes were kept from DATASET1 after removing markers in strong disequilibrium (*R*^2^ > 2). Linkage Disequilibrium (LD) pruning was done using PLINK software (Purcell et al., 2007). Data were converted from HapMap to numerical format using GAPIT3 software (Wang and Zhang, 2018) in R. DATASET1 was used in GWAS while DATASET2 was used to perform principal component analysis using “prcomp” function of “stats” package in R (R Core Team, 2020).

### Marker–Trait Association Tests and Candidate Gene Identification

Genome wide association analyses were performed with TASSEL software using Mixed linear model (MLM). The first three principal components calculated from LD pruned SNP dataset were included as a covariate in the MLM model to control for population structure. The MLM equation used in the analysis was:

Y=Xα+Pß+K+ε

Where: Y is the phenotype of a genotype; X is the fixed effect of the SNP; P is the fixed effect of the population structure; K is the random effect of the relative kinship; ε is the error term and is assumed to be normally distributed with a mean of zero. Percent change datasets calculated from BLUPs of traits measured under control and Al treatment trials were used as a phenotype input for the association analysis. Kinship matrix was also calculated using EMMA algorithm (Kang et al., 2010) and included in the model in addition to the PCs when using MLM in TASSEL. Manhattan plots and QQ plots were generated using the “CMPlot” R package (Lin-Yin, 2020) and significance levels were established using a Bonferroni correction at *p* < 0.05 based on the effective number of independent tests determined via SimpleM (Gao et al., 2008). We also used an exploratory significance cutoff at *p* < 0.0001. When reporting significant SNPs from each GWAS analysis, the SNP with the lowest *p*-value was chosen to represent each locus of interest. The significant SNPs were positioned to the *Phaseolus vulgaris* v.1 reference genome (G19833) using Jbrowse on Phytozome v.1.3 (Goodstein et al., 2012) to assess candidate genes in ± 100 k window positioning the significant SNP at the center. Gene annotation was done using mainly Phytozome v.1.3 but also other databases including TAIR, Pfam, KEGG, KOG, EXPASY, PANTHER were used to capture the maximum number of gene models. Literature was also consulted in addition to gene annotations to evaluate the function of the candidate genes.

## Results

### Differential Response of Genotypes to Al Treatment

The analysis of variance (ANOVA) was done on 227 genotypes remained after outlying genotypes were filtered out. The resulting analysis indicated the presence of significant variations (*p* < 0.001) among the genotypes for all traits under Al treated and control treatment experiments (Table 1). The small variance values of replication of treatments over time indicated that replication had little effect on the expression of each trait. This is because the greenhouse condition and the root zone conditions were monitored and maintained at similar conditions throughout the experiment (Supplementary Figure 2).

**TABLE 1:** Mean, Minimum (Min), Maximum (Max), genotypic variance (σ2g), replication variance (σ^2^r) error variance (σ^2^e) and heritability values of traits measured for the root phenotypes of 227 common bean genotypes from the Andean Diversity Panel (ADP) grown under Al treated and control hydroponic conditions.

Treatment	Trait	Mean	Min	Max	σ^2^g	σ^2^r	σ^2^e	H^2a^‡	Mean %Δ†	Min %Δ†	Max %Δ†
Al treatment	AvgD	0.92***	0.53	1.41	0.013	9.41×10^–5^	0.003	0.87	22.78	0.37	105.56
	NRF	363.86***	40	887	20986	6.19	1799.33	0.92	–66.43	–2.78	–97.22
	NRL	593.68***	82	1342	50931	1.2 × 10^–6^	2038.8	0.96	–67.83	–0.65	–97.25
	NRT	68.83***	9	153	437.21	0.55	65.99	0.87	–6.58	–94.69	–65.52
	RSA	20.45***	4.16	68.25	54.32	0.822	18.14	0.75	–65.27	–2.61	–93.24
	RV	0.47***	0.09	1.69	0.036	0.001	0.02	0.67	–56.58	–2.44	–93.03
	TRL	72.89***	7.73	200.7	699.44	0.122	788.4	0.89	–70.4	–0.92	–96.05
Control treatment	AvgD	0.76***	0.49	1.2	0.008	7.2 ×10^–4^	0.006	0.57			
	NRF	1366.45***	4	3519	365396	0.00	30730	0.92			
	NRL	2275.72***	11	5639	988320	0.00	52187	0.95			
	NRT	230.11***	4	550	7932.15	0.00	1296.2	0.86			
	RSA	69.48***	3.3	158.58	620.68	10.59	118.29	0.84			
	RV	1.36***	0.05	4.07	0.29	0.01	0.12	0.71			
	TRL	290.07***	11.9	638.31	11293	0.879	1043.34	0.92			

AvgD, average root diameter (mm); NRF, number of root forks; NRT, number of root tips; NRL, number of links; RSA, root surface area (cm^2^); RV, root volume (mm^3^); TRL, total root length (cm); ^‡^Broad sense Heritability calculated using the formula explained in Holland et al., 2003. ^†^Percent change of traits in aluminum treatment from control treatment. ***Means a significance level at p < 0.001.

Broad sense heritability for all traits were high in both experiments of Al and non-Al treatments (*h^2^* > 0.7) except for root volume (RV) in Al which was slightly lower (*h*^2^ = 0.67) and average root diameter (AvgD) in control (*h*^2^ = 0.57). Average total root length (TRL) (290.07 cm) was higher in control treatment while the range was narrower in Al treatment (7.7 to 200.7 cm) compared to the control treatment (11.9 to 638.3 cm). Similar trends were observed for root surface area (RSA), RV, number of root tips (NRT) and number of root forks (NRF). Unlike other traits, average AvgD (0.92 mm) was higher in Al treatment with narrow range (0.49 to 1.2) in control treatment compared to Al treatment (0.53 to 1.41 mm).

**FIGURE 1:**
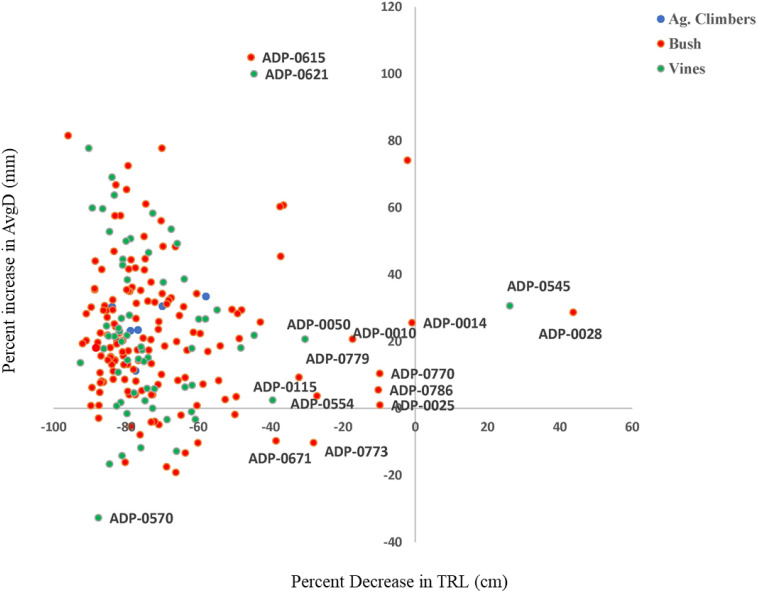
Identification of common bean genotypes from the Andean diversity panel (ADP) with low percent reduction of total root length and Low percent increase in average root diameter (ARD).

The treatment with aluminum had a pronounced effect in the quantitative traits measured. Higher average percent reduction was recorded for TRL (−75.18), RV (−64.96), RSA (−70.76), NRF (−73.56), NRT (−70.74), and NRL (−73.91) under Al treatment while percent increase was recorded on AvgD (+21.05) (Table 1 and Figure 1). In general, the heritability of traits in both treatments were high indicating the good repeatability of the hydroponic evaluation of traits. High heritability made the use of the averages for these traits as suitable for GWAS analysis. Also, treatment with aluminum had a pronounced effect on all the quantitative traits measured.

### GWAS and Candidate Gene Identification

**FIGURE 2:**
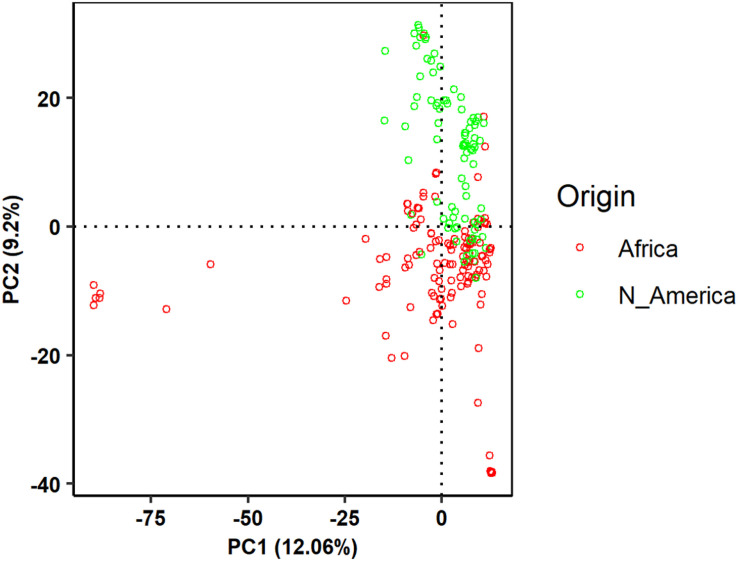
Principal component analysis for common bean genotypes from the Andean diversity panel (ADP) based on the single nucleotide polymorphism (SNP) dataset pruned for the criteria of non-linkage disequilibrium (LD).

**FIGURE 3:**
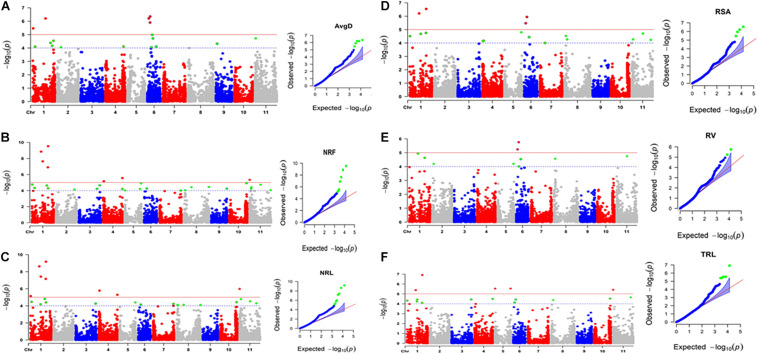
Manhattan and QQ plots of genome wide association study (GWAS) results of Average root diameter **(A)**, Number of root forks **(B)**, number of root links **(C)**, Root surface area **(D)**, Root volume **(E)**, and Total root length **(F)**, where single nucleotide polymorphism (SNP) loci are ordered by physical position and grouped by chromosome. The black dash line indicates the genome wide significance threshold. The SNP loci highlighted in red were significant with a given trait at *p*-values lower than the Bonferroni corrected significance cut-off value for number of independent tests which was *p* = 1 × 10^– 5^.

The broad sense heritability of each trait was high indicating the root traits measured in this study were suitable for GWAS analysis. The first two principal components accounted for 28.2% of the total variation (Figure 2) and were included as a covariate in GWAS analysis. GWAS associations identified multiple genomic regions associated with the six root traits (AvgD, NRF, NRL, RV, RSA, and TRL). Fifteen significantly associated SNPs resided on five chromosomes: Pv01, Pv04, Pv05, Pv06, and Pv11 at *p*-value = 1× 10^–5^ Bonferroni corrected *p*-value for independent number of tests (Figure 3 and Table 2). The strongest association was found for NRF and NRL with S1_38584873 and S1_25957702 resided on chromosome Pv01. For AvgD, five significant signals were identified on chromosome Pv01 and Pv06 and all explained 70% of the phenotypic variation.

**TABLE 2:** List of significant SNP markers detected for root traits evaluated for the common bean Andean Diversity Panel (ADP) showing physical position, association level (*p*-value), phenotypic variation (R^2^) explained by the locus, additive SNP effect and minor allele frequencies (MAF).

Trait	SNP	Chr.	SNP-Position	*p*-value	R^2^	SNP effect	MAF
AvgD	S1_30086531	Pv01	30086531	6.25E-07	0.15	–0.57	G (0.41)
AvgD	S1_1396792	Pv01	1396792	3.43E-06	0.14	0.81	G (−0.44)
AvgD	S6_6644197	Pv06	6644197	4.43E-07	0.13	–5.87	T (0.09)
AvgD	S6_3698197	Pv06	3698197	6.19E-07	0.14	–6.45	G (0.11)
AvgD	S6_6504245	Pv06	6504245	1.27E-06	0.14	–3.75	T (0.11)
NRF	S1_38584873	Pv01	38584873	2.99E-10	0.2	–11.23	T (0.15)
NRF	S1_22164004	Pv01	22164004	1.41E-09	0.19	7.18	C (0.17)
NRF	S1_25957702	Pv01	25957702	2.30E-08	0.16	11.09	A (0.19)
NRF	S1_38162226	Pv01	38162226	1.27E-07	0.13	–11.16	G (0.15)
NRF	S4_45347157	Pv04	45347157	2.76E-06	0.1	0.75	T (0.08)
NRF	S4_1455630	Pv04	1455630	6.82E-06	0.1	–3.33	T (0.34)
NRF	S11_44269	Pv11	44269	4.52E-06	0.1	–3.12	G (0.13)
NRL	S1_38584873	Pv01	38584873	6.67E-10	0.2	–10.36	T (0.15)
NRL	S1_22164004	Pv01	22164004	2.36E-09	0.19	6.64	C (0.17)
NRL	S1_25957702	Pv01	25957702	3.77E-08	0.16	10.17	A (0.19)
NRL	S1_38162226	Pv01	38162226	6.96E-08	0.13	–10.57	G (0.15)
NRL	S1_908352	Pv01	908352	7.20E-06	0.1	3.84	G (0.12)
NRL	S4_1455630	Pv04	1455630	1.65E-06	0.11	–4.1	T (0.34)
NRL	S4_45347157	Pv04	45347157	5.10E-06	0.1	1.78	T (0.08)
NRL	S11_44269	Pv11	44269	1.04E-06	0.12	–4.36	G (0.13)
RSA	S1_38584873	Pv01	38584873	2.87E-07	0.14	–7.32	T (0.15)
RSA	S1_22164004	Pv01	22164004	6.21E-07	0.14	3.8	C (0.17)
RSA	S6_6504245	Pv06	6504245	1.13E-06	0.14	3.52	T (0.11)
RSA	S6_3698197	Pv06	3698197	3.35E-06	0.12	1.58	G (0.11)
RV	S6_6504245	Pv06	6504245	1.75E-06	0.14	4.77	T (0.11)
RV	S6_3698197	Pv06	3698197	5.74E-06	0.12	2.03	G (0.11)
TRL	S1_38584873	Pv01	38584873	1.22E-07	0.15	–7.18	T (0.15)
TRL	S1_22164004	Pv01	22164004	4.12E-06	0.12	3.92	C (0.17)
TRL	S5_39203550	Pv05	39203550	2.84E-06	0.1	0.84	T (0.28)
TRL	S5_736525	Pv05	736525	2.88E-06	0.1	2.92	G (0.31)
TRL	S11_44269	Pv11	44269	3.80E-06	0.1	–1.44	G (0.13)

*TRL, total root length (cm); RSA, root surface area (cm^2^); RV, root volume (mm^3^); AvdG, Average root diameter (mm); NRT, number of root tips; NRF, number of root forks; NRC, number of root crosses; NRL, number of links.*

The largest number of significant signals (8 SNPs) were found for NRL on chromosomes Pv01, Pv04, and Pv11. However, most of them (5 SNPs) were from Pv01. Collectively, the eight SNP loci accounted for 91% of the variation in NRL. Same signals (7 out of 8 SNPs) were also found for NRF, where they explained 98% of phenotypic variation. For RSA, four significant SNP signals were located on chromosomes Pv01 and Pv06. Similarly, two SNP loci on Pv06 were significant for RV. Two of the significant SNP (S6_6504245 and S6_3698197) signals on chromosome Pv06 were common for AvgD, RSA and RV. For TRL, five significant SNPs were identified on three chromosomes (Pv01, Pv05, and Pv11) where they accounted for 57% of the phenotypic variation. From this description we saw that there were several SNPs associated with more than one trait. The list of significant SNP loci and their association level, the phenotypic variation explained by significant markers are indicated in Table 2 and visualized in Figure 2.

Based on significant GWAS hits and the sequence of the common bean reference genome (G19833) scanned with ±100 kb window from each SNP, we identified 192 gene regions of interest. The gene ID, Phytozome-annotated function of the gene, genomic position, and top or bottom chromosomal strand direction of the gene on the genome are presented in Supplementary Table 2. The function of these genes fell in the categories of regulation of plant development, signal transduction and reception, carbohydrate metabolism, integral components of membranes, binding protein and other processes. Each significant marker produced multiple hits of coding regions making identification of genes difficult. For the sake of simplicity, we report genes that were meaningful with regards to Al stress tolerance rather than all genes in the GWAS window.

Nearly half (48%) of the identified genes were associated with seven significant markers on chromosome Pv01. AvgD was associated with two SNP loci (S1_30086531 and S1_1396792) on chromosome Pv01. These two loci were linked with many candidate genes. Among these, *Phvul.01G117300* which was found ∼100 kb downstream of the significant SNP S1_30086531. Another gene associated with same marker and found 38 kb downstream was *Phvul.01G117800*. Four genes were found in close vicinity of the second SNP substantially associated with AvgD. While *Phvul.001G017000*, and *Phvul.001G017400* were located 72 and 39 kb downstream, *Phvul.001G017800*, and *Phvul.001G018000* were located upstream at 29 and 35.5 kb, respectively, of the SNP S1_1396792.

Four root traits namely, NRF, NRL, RSA, and TRL were significantly associated with two SNP loci (S1_22164004 and S1_38584873) on chromosome Pv01. The SNP S1_38584873 was a highly significant marker explaining 20, 20, 14, and 15% of the phenotypic variation in NRF, NRL, RSA, and TRL, respectively. We found 16 genes located 100 kb up and downstream of S1_38584873. Among these genes, *Phvul.001G141900* and were involved in abiotic stress tolerance. *Phvul.001G141900* was found 98.5 kb downstream and *Phvul.001G142750* was found in the region containing the significant marker itself. Another five genes (*Phvul.001G139000, Phvul.001G139100, Phvul.001G139200, Phvul.001G139250, Phvul.001G139400*, and *Phvul.001G139600*) were also found within 91 kb distance downstream of SNP S1_38162226, a significant SNP associated with NRF and NRL.

Fewer candidate genes were found on other chromosomes apart from Pv01 and we reported them in consecutive order. On chromosome Pv04, NRF and NRL were associated with two significant SNP loci; S4_45347157 and S4_1455630. Two genes *Phvul.004G150600* and *Phvul.004G151200* were located downstream at 81 and 35 kb, respectively, of SNP S4_45347157. Another SNP, S4_1455630, was surrounded by multiple genes which encode leucine-rich repeat-containing protein. On chromosome Pv05, 10 genes encoding leucine-rich protein kinase related proteins and leucine-rich repeat containing proteins were found surrounding only one significant SNP (S5_39203550). This SNP is significantly associate with TRL and accounted 10% of the total phenotypic variation explained in TRL. Chromosome Pv06 had one candidate gene, *Phvul.006G014600*, 63.5 kb upstream, 59 kb upstream of a significant SNP S6_6644197 which was significantly associated with three traits; AvgD, RSA, and RV, encoding a Zinc-binding protein. Finally, on chromosome Pv11, we found only one significant SNP associated with TRL. A total of 16 functional genes were identified near the significant SNP S11_44269. Among these genes, *Phvul.011G000700* and *Phvul.011G001300* were located 2 and 65 kb upstream of the significant SNP.

## Discussion

The root systems of beans are the major interface between these plants and numerous biotic and abiotic factors and enables them to avoid these environmental challenges by sensing and responding to them (Rao et al., 2008). Roots are used by plants to absorb water and nutrients from the soil, to store food or nutrients and used as architecture to anchor plants to the ground (Kochian et al., 2004). Therefore, roots must be protected from stresses such as rhizotoxicity of Al which causes inhibition of root growth and function (Horst et al., 2009). Genetic and environmental factors impact both the structure and functions of roots under Al stress (Rangel et al., 2010). Understanding the pattern and magnitude of traits relations, identifying breeding objectives, assessing the available genetic diversity, and identifying candidate genes are the most important tasks of a breeding program when considering root improvement in beans.

Andean beans are important components of agri-food system in Africa and Latin America where they are preferred by consumers because of their large seeds and colors. Andean beans are produced by small holder farmers on marginal lands where acidic soils are more prevalent (Blair et al., 2009) and productivity is constrained by Al toxicity and other abiotic stressed including drought. Andean beans are characterized by narrower genetic diversity (Cichy et al., 2015a) and their genetic improvement has lagged behind Mesoamerican bean improvement. Some promising results show that it is possible to improve aluminum tolerance in Andean beans (Blair et al., 2009; López-Marín et al., 2009).

The use of hydroponics to study aluminum resistance in plants is an option to complement field evaluations. Screening in hydroponics has advantages over field experiments in allowing the evaluation of large number of genotypes and providing precise control over the timing and concentration of nutrient supply and Al stress (Butare et al., 2011). Evaluation of genotypes in hydroponics experiments has been used for determining aluminum tolerance expression traits in many plants; including, barley (Ma et al., 1997), wheat (Sasaki et al., 2004, 2010; Furuichi et al., 2010), sorghum (Magalhaes et al., 2004), soybeans (Bianchi-Hall et al., 2000), and common beans (Butare et al., 2011).

Our study assessed the genetic variation in root traits of 227 Andean common bean genotypes under contrasting Al toxicity treatments (control with no Al treatment versus 50 μM Al toxicity treatment) which produced additional insights helpful for the development of new improved varieties with greater adaptation to problematic Al toxic soils. The greenhouse conditions of tank temperature, electrical conductivity, and pH around the root of the bean seedlings were monitored in real time and kept constant to avoid external variation (Supplementary Figure 1). The Al toxicity concentration used was ideal for revealing genotypic differences for root traits considered in this study. A higher percentage of reduction was observed in all root traits measured under Al toxicity treatment except average root diameter which showed an increase (23% on average) but with a wide range in variation (0.37 to 105.56%). Previous hydroponic studies with common bean created consistent levels of Al toxicity for germplasm screening as we did here; however, we added a level of precision in temperature and pH control through constant, real-time, in-tank monitoring.

The higher percentage reduction in root traits other than average diameter indicated that inhibition of root elongation, lateral root initiation and outgrowth and increased root diameter are important effects of Al toxicity in common beans. The increase in average root diameter and reduction in total root length under Al- toxicity treatments were also reported by various authors (Blair et al., 2009; Butare et al., 2012). Studies suggested that genotypes with lower percent inhibition of total root length and percent increase of average root diameter were most Al tolerant (Rao et al., 2016, 2008). Several genotypes (e.g., ADP-014, ADP-028, and ADP-545) had an increased percentage of total root length and average root diameter (up to 40%) and considered as moderately tolerant to Al toxicity. Blair et al. (2009) and Butare et al. (2012, 2011) identified common bean genotypes from an Andean gene pool and interspecific inbred lines of *Phaseolus* species that are tolerant to a higher level of Al concentration.

The Al tolerance we reported in this study could be due to different reasons. Tolerant plants developed two different physiological mechanisms to Al toxicity. The mechanisms of Al toxicity tolerance studied in many plant species identified both external and internal plant mechanisms (Hede et al., 2001). The external mechanism is where plants exclude Al from the root apex using selective permeability of the plasma membrane, exudation of chelating organic acids, production of root mucilage and exudation of root phosphate. The internal tolerance mechanisms that confer the ability to tolerate Al in the plant symplasm usually involves Al binding proteins (Gupta et al., 2013). These mechanisms are related to mitochondrial metabolism and acid transport (Nunes-Nesi et al., 2014).

Generally, common bean is relatively poor at adapting to Al stress conditions (Rao et al., 2008). However, studies reported that some Al tolerant common bean genotypes display Al activated exudation of citrate and Al chelating organic compound (Mugai et al., 2000; Eticha et al., 2010; Rangel et al., 2010) which is much smaller in the Al sensitive genotypes than in tolerant ones (Miyasaka et al., 1991). The exudation of citrate and Al chelating organic compounds help the plant to exclude Al from their root system. Recent advances in physiological, biochemical and molecular studies also revealed that the modification of the binding properties of the root apoplast contributes to Al tolerance (Horst et al., 2010). Testing of top lines from the hydroponic trials for analysis of organic acid exudation *in vitro* or on acid soils, would be useful.

Genome wide association study exploits the linkage disequilibrium (LD) present among individuals from natural populations or germplasm collections to dissect the genetic basis of complex quantitative trait variation with a powerful resolution as compared to studying a biparental mapping population. Our GWAS study allowed us to identify 15 significant SNP loci associated with six root traits on five common bean chromosomes Pv01, Pv04, Pv05, Pv06, and Pv11. Seven of the 15 significant markers for all traits other than RV were located on chromosome Pv01. Furthermore, the majority of Al stress tolerance related genes were also on Pv01. This suggested that Pv01 be considered for targeted study to further understand the mechanism of Al tolerance and associated metabolic pathways and network of genes in common beans. We found no significant SNP associated with any of the trait studied on chromosome Pv09. In contrary López-Marín et al. (2009) found Al responsive QTL (*Trl9.1*) on this chromosome derived from G19833 which was not included in the current study. Njobvu et al. (2020) identified Al tolerance QTLs on chromosomes Pv02, Pv07, Pv09, and Pv10, chromosomes on which we did not find significant SNP markers. However, they positively identified QTLs on chromosomes Pv04, Pv05, and Pv 06, where we found many functional genes associated with plant responses against Al toxicity.

Related to the QTLs, the candidate genes identified in this study can be classified in to the following categories: genes encoding malate transporters, MATE transporters, protein kinases, receptors, and growth regulators, or pentatricopeptide proteins (PPRs). One of the most important genes identified was *Phvul.01G117300* which encoded an Al activated malate transporter homologous to the gene *ALMT1* (Hoekenga et al., 2006), a protein involved in malate exudation (Liu et al., 2012; Kobayashi et al., 2013, 2007). This gene was found on chromosome Pv01 and had similar sequence to *Phvul.007G025900*, a gene found on chromosome Pv07 found influencing Al tolerance in common bean by Njobvu et al. (2020). A homolog from Al tolerant wheat lines, also encoding membrane protein (*TaALMT1*) and facilitating malate efflux was reported to be highly expressed in this cereal’s root apices (Ryan et al., 2009). This confirmed work in Arabidopsis, where *AtALMT1* was identified as critical for Al tolerance (Hoekenga et al., 2006). Study of association of aluminum tolerance candidate genes in beans with the balance of organic acids released by roots could be done through gene and metabolic expression profiles.

Secondly, a multidrug and toxic compound extrusion *(PvMATE)* membrane protein gene, (*Phvul.001G017400*) was identified for Al tolerance in bean. The MATE efflux proteins are important to Al tolerance (Wu et al., 2019) and *HvMATE* was first described as a candidate for controlling Al tolerance in barley by Wang et al. (2007). Ryan et al. (2009) showed correlation between the expressed sequence for *TaMATE* and citrate efflux in Al tolerant wheat cultivars describing organic acid exudation as an important mechanism of Al tolerance along with genes involved in Al detoxification through iron translocation. The *PvMATE* gene we found is a functional homolog of genes characterized in sorghum (Magalhaes et al., 2004, 2007), barley (Zhou et al., 2013), maize (Maron et al., 2010) and soybean (Liu et al., 2016). Successful cloning of Al tolerance QTL was reported using this gene in sorghum and barley (Liu et al., 2014). Among other genes identified for Al tolerance was *Phvul.01G117800*, encoding a homolog of phosphatidylinositol 4-kinase gamma 1 (PI4K), which in Arabidopsis activates early Al signaling and regulates the process of Al-induced malate transport by *AtALMT1*. *Phvul.001G142750* was another gene which encoded a cation efflux protein, involved in transmembrane transport of cations that confer metal tolerance (Mäser et al., 2001; Singh et al., 2016).

A third group of genes we found involved in Al tolerance were classified for signal reception and growth regulation (*Phvul.001G017000* and *Phvul.001G018000*). Two genes encoded threonine protein kinases which are central in signal transduction from receptors that sense environmental conditions into appropriate outputs such as shifts in metabolism, gene expression, and cell growth or division (Hardie, 1999). *Phvul.004G151200* was another candidate gene identified by this study, which encoded a mitogen-activated protein kinase, also for signaling and DNA repair during and after Al-induced nuclear damage thus providing an adaptive response in root cells (Panda and Achary, 2014). Many additional genes were further candidates which encoded leucine-rich repeat (LRR)-containing proteins on chromosome Pv04 and 05 (Supplementary Table 2). These genes could be important in the elongation zone of root meristem and involved in cellular proliferation, plant growth and stress tolerance (Jones and Jones, 1997; Lorenzo et al., 2009; Zhao et al., 2018). Additionally, we found *Phvul.001G017800*, a gene encoding a cytochrome P450 enzyme that might be important to abiotic stress responses (Pandian et al., 2020) and *Phvul.001G141900*, a gene for a zinc finger CCCH domain-containing proteins, found to be involved in salt and drought tolerance (Sun et al., 2007; Bogamuwa and Jang, 2016).

Finally, a fourth class of genes were PPPs, whose involvement in Al tolerance is probably new to plant science. Genes *Phvul.011G000700* and *Phvul.011G001300* were examples of this class. These genes are likely to encode pentatricopeptide repeat (PPR) proteins known to be involved in post-transcriptional regulation of gene expression (Kosová et al., 2018) during abiotic stresses like drought and salinity (Xing et al., 2018; Sertse et al., 2019). The finding was supported by the fact that we also found many genes associated with salt and drought tolerance. This showed the possible cross talk and links of tolerance mechanisms between different abiotic stresses. Similar reports support this result in common bean for abiotic stresses of the acid soil complex of Al toxicity plus low P availability (López-Marín et al., 2009; Njobvu et al., 2020). In general, Al tolerance in common beans is complex and governed by coordination of many genes involved in many metabolic pathways from signal transduction (Canonne et al., 2011; Panda and Achary, 2014) to transcription and translation, post-transcriptional regulation of gene expression (Kosová et al., 2018), transmembrane transport of metabolites (Liu et al., 2016; Singh et al., 2016), and cell proliferation and plant growth regulation (Lorenzo et al., 2009).

In conclusion, this study identified sources of genetic variation for Al tolerance, estimated heritability of root characteristics in response to Al stress, and explained complexity of root reactions to Al in common beans. Significant association between SNP markers and root traits were found with multiple QTL explaining polygenic inheritance involving four chromosomes. Putative candidate genes related to malate and citrate exudation were encountered near significant SNP loci. Similar studies in wheat also showed complex nature of Al tolerance (Ryan et al., 2009, 2010; Raman et al., 2010). Further to this work we identified a number of candidate genes, whose regulation and detailed function in associated pathways could be studied in the future as they relate to Al tolerance. The utilization of germplasm sources, genetic diversity and genes identified in this research will hopefully permit the improvement of common bean varieties tolerant to Al toxicity and other related abiotic stresses. The positive GWAS loci and flanking SNP markers will enable common bean breeding programs to make rapid genetic gains for a difficult to assess root trait that would be underground or would require hydroponic growth systems for phenotyping. Candidate genes found in this study could be converted into direct gene markers for the specific traits of interest after thorough examination of the nucleotide variation at their location and implications for marker development in bean germplasm.

## Data Availability Statement

The original contributions presented in the study are included in the article/Supplementary Material, further inquiries can be directed to the corresponding author.

## Author Contributions

MB and DA planned the study, setup the experiment, took data, and wrote the manuscript. DA organized and analyzed the data and prepared graphs and tables. Both authors contributed to the article and approved the submitted version.

## Conflict of Interest

The authors declare that the research was conducted in the absence of any commercial or financial relationships that could be construed as a potential conflict of interest.

## Footnotes

1 http://arsftfbean.uprm.edu/bean/?p=472
