# Supplementary figures and images for: Genome Wide Association Mapping of Root Traits in the Andean Genepool of Common Bean (Phaseolus vulgaris L.) Grown With and Without Aluminum Toxicity
Source: Front Plant Sci. 2021 Jun 25;12:628687. doi: 10.3389/fpls.2021.628687 (PMC8269929; doi:10.3389/fpls.2021.628687)

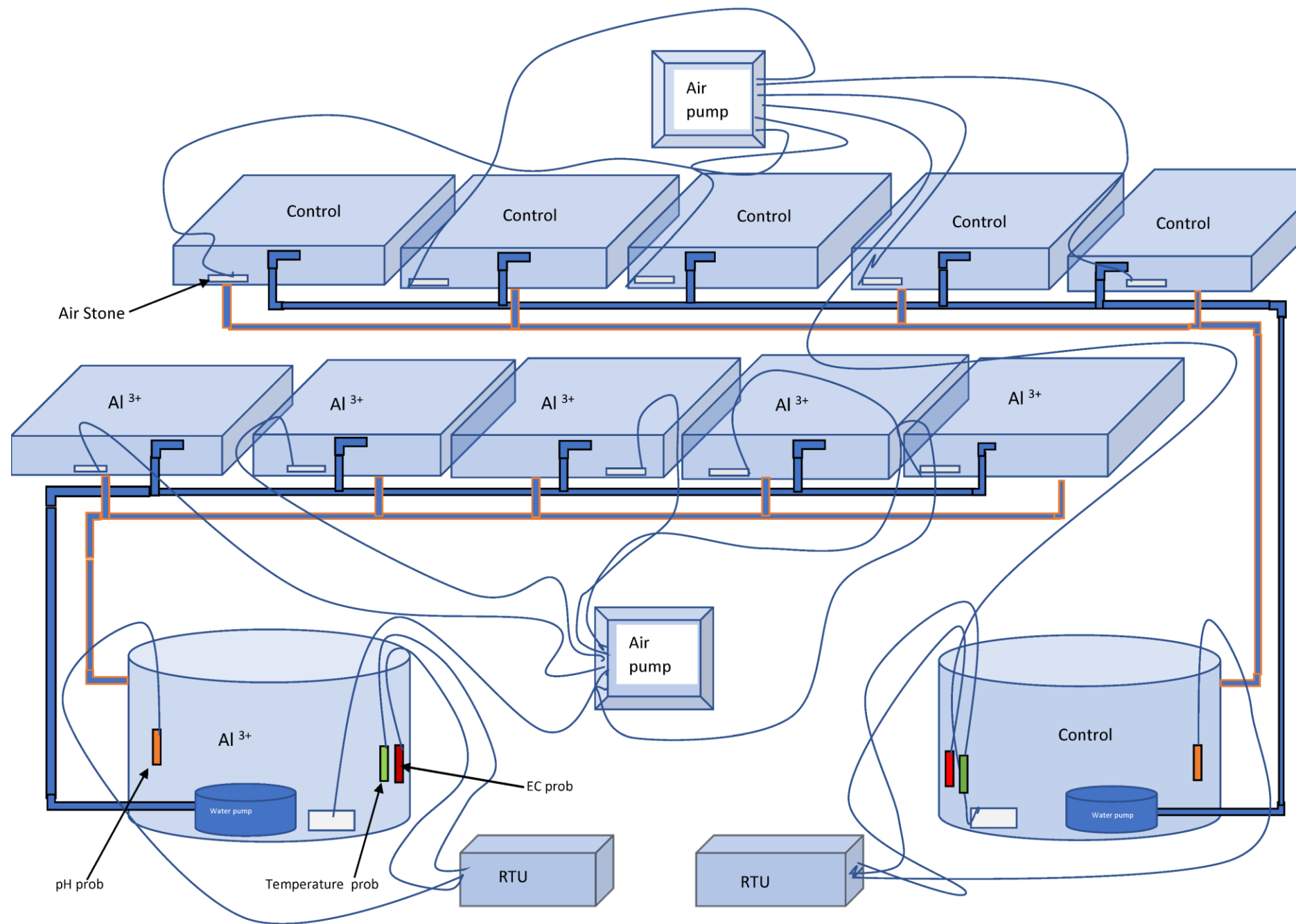

Supplement: Supplementary file 1 [file Image_1.pdf]

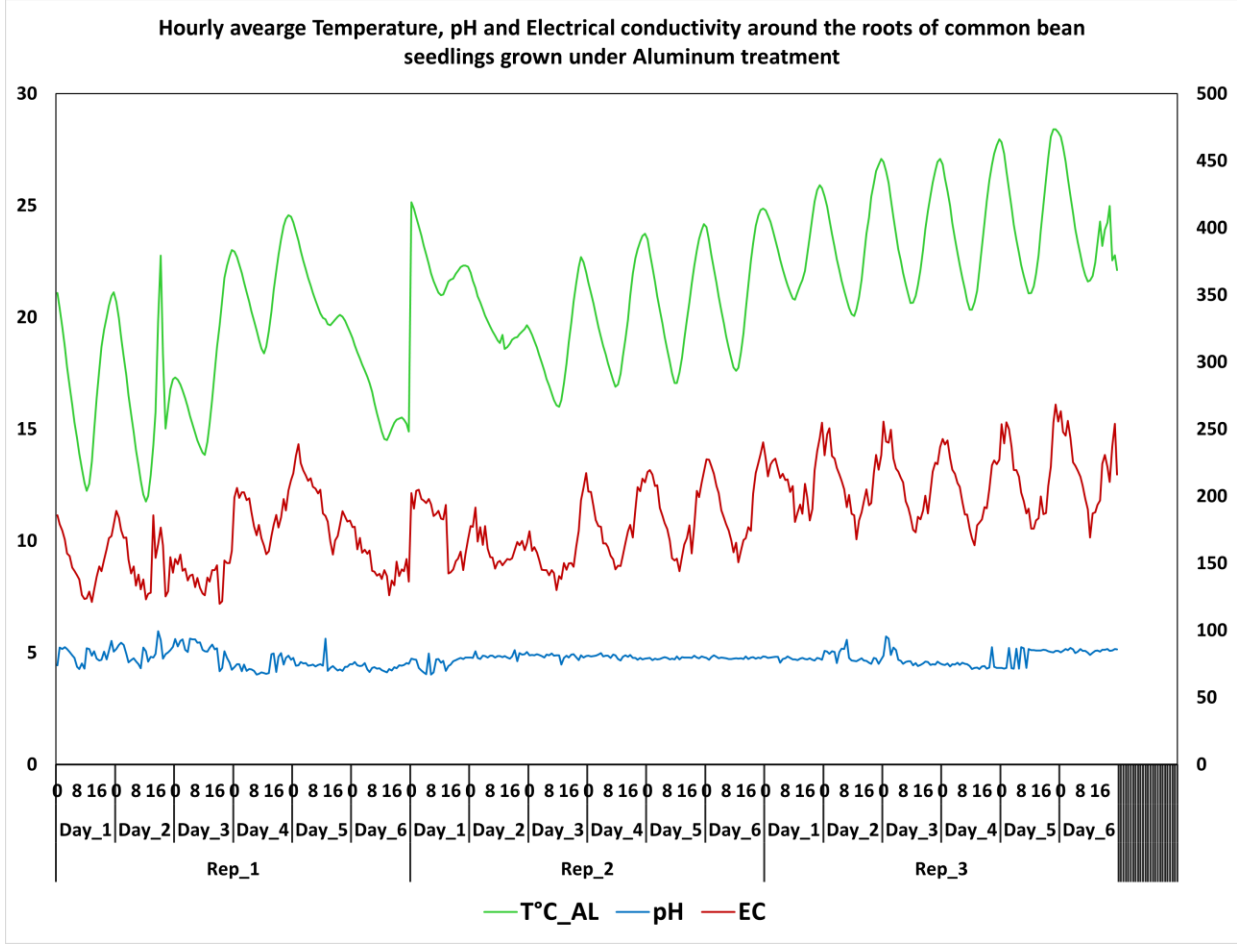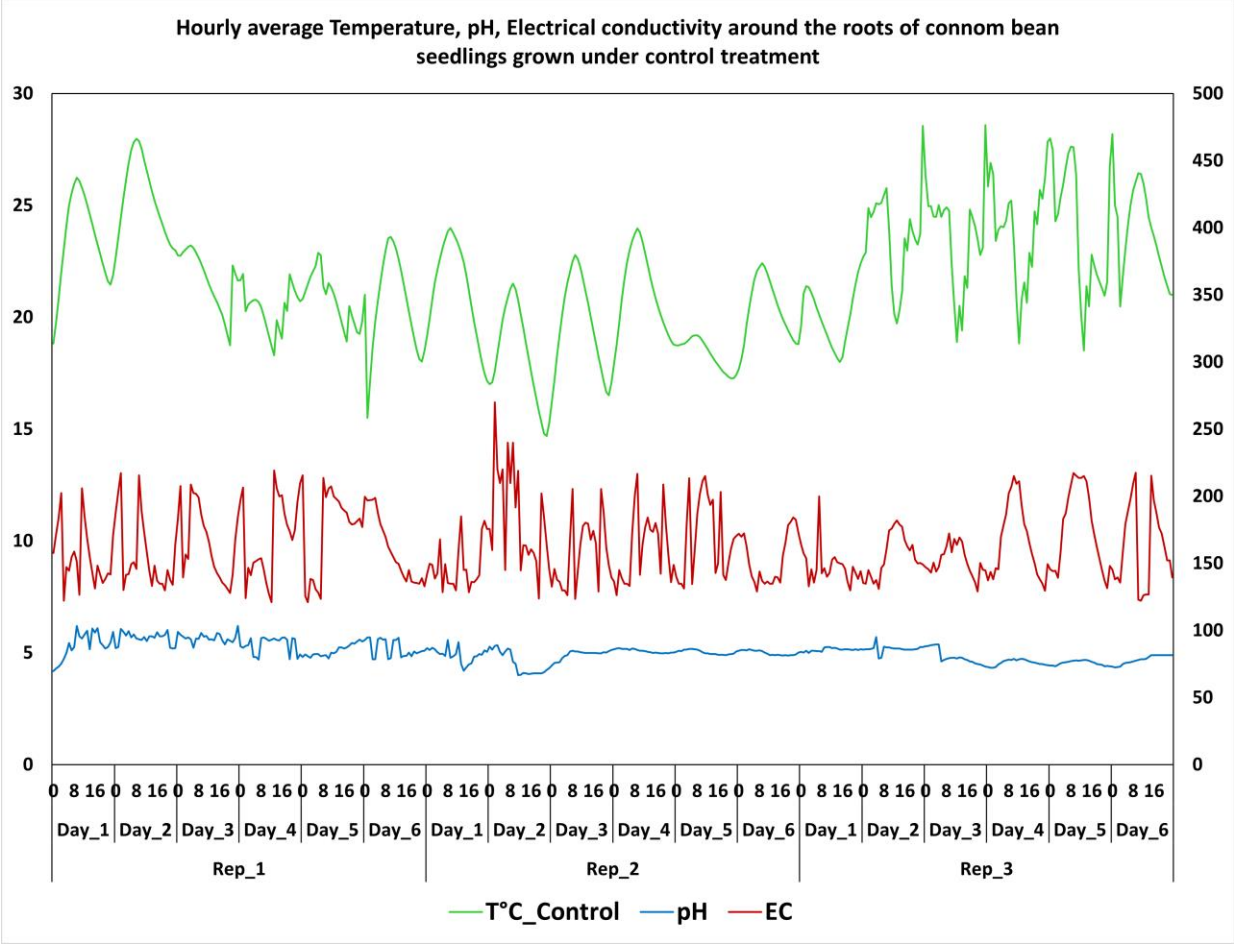

Supplement: Supplementary file 2 [file Image_2.pdf]
